# Substance use disorder and the baby boom generation: Does Berlin outpatient addiction care face a sustained change?

**DOI:** 10.1111/dar.13245

**Published:** 2021-01-28

**Authors:** Sara Specht, Barbara Braun‐Michl, Larissa Schwarzkopf, Daniela Piontek, Nicki‐Nils Seitz, Manfred Wildner, Ludwig Kraus

**Affiliations:** ^1^ IFT Institut für Therapieforschung Munich Germany; ^2^ Pettenkofer School of Public Health, Ludwig‐Maximilians‐University Munich Germany; ^3^ Bavarian Health and Food Safety Authority Oberschleißheim Germany; ^4^ Department of Public Health Science, Centre for Social Research on Alcohol and Drugs Stockholm University Stockholm Sweden; ^5^ Institute of Psychology ELTE Eötvös Loránd University Budapest Hungary

**Keywords:** addiction care, cohort effect, baby boomers, alcohol and illicit substance use disorder, comorbid substance use disorders

## Abstract

**Introduction:**

The ageing of baby boomers is expected to confront addiction care with new challenges. This cohort had greater exposure to psychoactive substances in youth than earlier cohorts. In this study, we aimed to investigate whether Berlin addiction care is confronted with a sustained change in its clientele initiated by the baby boomers.

**Methods:**

Using data from Berlin outpatient addiction care facilities, we contrasted type of primary substance use disorder and number of comorbid substance use disorders in baby boomers with an earlier and a later cohort. To isolate cohort effects, two‐level random intercept regression models were applied in the overlapping age groups of the baby boomer cohort with each of the other cohorts.

**Results:**

Compared with the earlier cohort, alcohol use disorder lost importance whereas illicit substance use disorder gained importance in the baby boomers. Baby boomers presented a higher number of comorbid substance use disorders than the earlier cohort. Comparing baby boomers with the later cohort, these relationships pointed in the opposite direction.

**Discussion and Conclusions:**

Outpatient addiction care faces a sustained change to more illicit and comorbid substance use disorders. With increasing life expectancy and the ageing of baby boomers marked by higher substance use than previous cohorts, older clients, who had been under‐represented in outpatient addiction care, will gain relevance. Hence, addiction care has to adapt its offers to appropriately meet the changing needs of its clientele.

## Background

Baby boomers are the birth cohorts with increased birth rates after World War II. In Germany, the baby boom took place between 1954 and 1969 [1]. Emerging wealth formed them as a generation of self‐expression and pleasure in contrast to previous generations in Western countries [2, 3, 4]. Compared with earlier cohorts, baby boomers had greater exposure to alcohol, tobacco and illicit substances in youth [5], developed a higher acceptance towards drug consumption [6] and, as a result, are more susceptible to substance use [5]. Overall, these historical conditions have the potential to influence members of a cohort, which could then play a part in social changing processes [7, 8].

With the presence of baby boomers in addiction care, the primary problem for which individuals seek care is expected to shift; compared with earlier cohorts, a decline in the relative relevance of alcohol use disorder (AUD) and an increase in the importance of illicit substance use disorder (ISUD) is anticipated [5, 9, 10, 11, 12]. Individuals seeking help in addiction care primary for alcohol problems differ substantially from those with primary problems related to the use of illicit substances [13] and, subsequently, mark different fields of care.

The suggested upheaval in primary AUD to ISUD diagnoses in baby boomers may be different for women than for men. Despite an ongoing convergence of well‐established sex‐specific roles [14], sex‐specific socialisation processes could lead to temporally shifted cohort effects.

Regarding developments in mental health, there is evidence that later cohorts are burdened with a combination of multiple substance use disorders more often than earlier cohorts [11, 15]. Based on this, baby boomers are expected to qualify for more comorbid substance use disorders (CUD) than earlier cohorts, which in turn is linked to a higher overall burden and complicates treatment [16, 17, 18].

The majority of studies on baby boomer‐specific characteristics in addiction come from the USA where legislation, economic and cultural developments may differ, and the baby boom commenced earlier than in Germany [3]. However, similar trends can be expected in all high‐income countries because baby boomers were exposed to a similar youth culture that differed from that of their antecedents [4].

As the baby boomer cohort started to reach age 55 years and above from 2010 in Germany, the share of older age groups—which have been under‐represented in addiction care so far—is anticipated to rise as projected in other countries [19, 20, 21]. Considering the size, the relatively high life expectancy and the specific substance‐related use patterns of this cohort, the baby boomers are an important target group in addiction care, especially when it comes to the older age group. Hence, to facilitate addiction care planning, knowledge about baby boomers and the developments they will set off in the addiction care system is required. In this regard, comparing baby boomers against both an earlier and a later cohort will contribute to a better understanding whether baby boomers mark the beginning of a continuous change of substance‐specific help‐seeking in outpatient addiction care.

To close the knowledge gap on whether there had been a shift in cohort characteristics in German outpatient addiction care this study aims to examine: (i) whether outpatient addiction care seekers from the baby boomer cohort are less likely to be diagnosed with primary AUD and (ii) more likely to be diagnosed with ISUD than an earlier cohort; (iii) whether cohort effects in the likelihood of being diagnosed with AUD or ISUD differ by sex; (iv) whether baby boomers have more CUDs than an earlier cohort; and (v) whether characteristics observed in the baby boomers continue in a later cohort.

## Methods

### Setting and design

We analysed data from the outpatient Berlin Addiction Care Statistical Service, which contains a case‐based documentation of services in facilities of the type ‘outpatient counselling and treatment’ of the German‐wide standardised core dataset [22]. About 88% of the service provided in the facilities included in the dataset was outpatient addiction counselling and approximately 4% outpatient detoxification. Hereafter, the term ‘outpatient addiction care’ is used for these facilities. In case of several individual help‐seeking episodes, only the first episode was included. Data were entered by addiction care personnel. The documentation served as a guideline for addressing all relevant issues and contained admission and cessation variables. For comparability reasons, annual data for the years 2008–2016, where an identical set of variables was collected, were used. The annual participation rate ranged from 73% to 84% of all registered Berlin outpatient counselling and treatment facilities in the years 2012–2016 (participation rates before 2012 are not available). Data collection was performed in accordance with the ethical standards of the Helsinki Declaration as revised in 2013 and in accordance with regional (Berlin data protection law), national (German data protection law) and international (European General Data Protection Regulation) data protection requirements. Informed consent was obtained from all patients by the data collection facilities. Participation was not linked to any benefits or the selection of treatment offers.

### Variables of interest

Primary and further substance‐related diagnoses were coded according to the International Statistical Classification of Diseases and Related Health Problems, 10th revision, German Modification and refer to the 12 months before admission (mostly dependence but also harmful use). Information on diagnoses came from either prior diagnostics, assessment by addiction care personnel with expertise to make diagnoses or a clinician. The primary diagnosis was the addiction‐related diagnosis an individual sought help for. ISUD comprised disorders related to the use of opioids, cannabis, cocaine, stimulants, hallucinogens, volatile solvents or other psychotropic substances. CUDs burden reflected a sum score of positive diagnoses on F10–F19 (mental and behavioural disorders due to psychoactive substance use) and F55.0–F55.2 (abuse of the non‐dependence‐producing substances antidepressants, laxatives and analgesics). F55.0–F55.2 diagnoses were included to cover both dimensions of substance‐related problems (substance use disorder and abuse of particular medicaments). Cohort membership was defined by year of birth and comprised the following categories: ‘baby boomers’ born between 1954 and 1969 (annual birth rates above 1.1 million), the ‘earlier cohort’ born between 1938 and 1953, the ‘later cohort’ born between 1970 and 1985.

Age at admission was measured in years. Relationship with a partner was dichotomised into having a stable relationship or not. The level of school education was assessed by means of German degrees. A ‘low’ school education corresponds to a lower secondary school certificate or less (school attendance ≤9 years) and ‘at least middle’ school education to an upper secondary school certificate or higher (≥10 years of school education). The number of contacts during treatment was documented as well as whether someone had ever used any kind of addiction care service before.

### Analyses

Due to concerns regarding multicollinearity of age and cohort, age at admission was not handled as a control variable [for a discussion, see Ref. 23]. Instead, analyses were restricted to overlapping age groups of baby boomers and earlier cohort and baby boomers and later cohort, respectively. Two disjunct subsamples, one comprising members of the earlier and the baby boomer cohort at ages 55–62 (subsample 1) and one containing members of the later and baby boomer cohort at ages 39–46 (subsample 2), were created. All analyses were performed in each of the two subsamples. For an overall characterisation, the subsample‐specific cohorts were analysed descriptively regarding sociodemographic‐, disorder‐ and treatment‐related characteristics.

Controlling for time variations by including year of data collection as independent variable together with cohort membership in the overlapping age groups would have caused multicollinearity problems [23]. Thus, we applied multilevel regression to account for the nested structure of the data using year of data collection as the level 2 unit. Age at admission was considered by analysing the cohorts in the overlapping age groups. Consequently, the cohort effects are not attributable to age differences between the cohorts. In a sensitivity analysis (SA1), we compared the cohorts across the whole age range at hand. Here, we applied the same multilevel approach, but kept age as a grouped level 1 control variable (overall sample) to explore whether the cohort differences persisted in a larger age range.

Two‐level random intercept logistic regression models were applied to test cohort differences using ‘AUD’ and ‘ISUD’ as outcome variables. Models with primary diagnoses of opioids, cannabis and stimulants/cocaine use disorder were run as sensitivity analyses (SA2) to test whether the results differed from the overall model with ISUD as outcome. The number of CUDs was examined by means of two‐level random intercept Poisson regression models and results were presented as incidence rate ratios [24].

By calculating the intraclass correlation coefficient in the empty random intercept models for each outcome and conducting a likelihood ratio test, we assessed whether using a random intercept model helped to explain the variance in the outcomes [24, 25]. For the outcome ‘number of CUDs’ in subsample 2, the pre‐analyses indicated use of a single intercept model. To take time effects into account nevertheless, year of data collection was kept as a second‐level unit. Potential covariates were selected based on previous knowledge stemming from the annual report of the German and the Berlin Addiction Care Statistical Service [13, 26], and were further investigated in exploratory pre‐analyses to examine if they had significant associations with the outcomes. This comprised sex, relationship with a partner, school education, kind of primary diagnosis (not in AUD/ISUD models), number of contacts during treatment and prior utilisation of addiction care (not in AUD/ISUD models). Building on the empty random intercept models, further models were estimated including these preselected covariates as additional level 1 variables (fixed effects). To decide about inclusion, variables were entered one at a time and compared with the empty random intercept model [24].

In all six final models (AUD, ISUD and CUDs for each subsample), besides cohort and sex as predetermined variables, the level of school education and partnership were included as level 1 covariates. Except for ISUD in subsample 1, this applied to the number of contacts during treatment too. In the Poisson models of the number of CUDs, the kind of primary diagnosis and former utilisation of addiction care were added as additional covariates. To investigate the existence of sex‐specific cohort effects for AUD and ISUD, a cohort*sex interaction was included in the respective models. Following models were compared [27]: a model with both variables on level 1 and a model containing the interaction term plus cohort and sex. We defined the model with the lower Bayesian information criterion as the better fitting one. For AUD (1) and ISUD (2), it was the model without interaction. The same applied to the sensitivity analyses with cannabis and stimulants/cocaine use disorder. For primary opioids use disorder in subsample 1, it was the model with the interaction term.

All analyses were conducted with Stata/SE 15 (Stata Corp LP; College Station, TX, USA). An alpha level of 0.05 was used for statistical tests.

## Results

### Samples' descriptions

**Figure 1:**
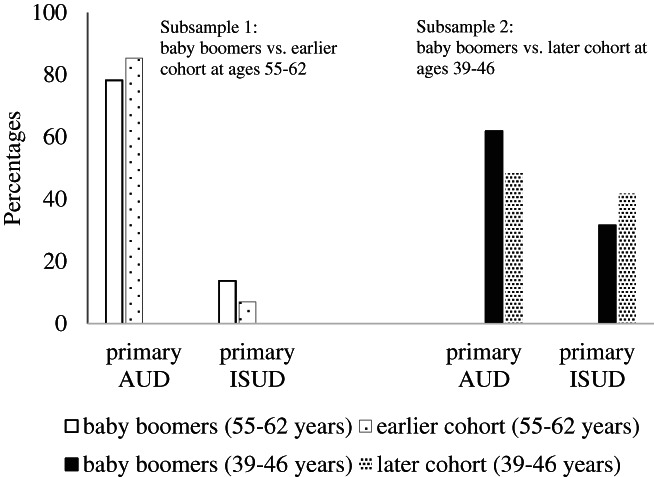
Cohort differences. AUD, alcohol use disorder; ISUD, illicit substance use disorder. The primary diagnosis is the addiction‐related diagnosis an individual sought help for.

Subsample 1 consisted of 6524 cases, comprising 64.2% baby boomers. The percentage of women was higher in the earlier cohort (34.0%) than in the baby boomers (30.2%). The most frequent primary diagnosis was AUD with 85.4% in the earlier and 78.2% in the baby boomer cohort. Members of the earlier cohort were on average diagnosed with 1.3 CUDs (*SD* = 0.7), whereas baby boomers had 1.5 diagnoses (*SD* = 1.0) (Table 1, Figure 1).

**Table 1:** Cohort characteristics

Characteristics	Subsample 1: Baby boomers vs. earlier cohort				Subsample 2: Baby boomers vs. later cohort				
	Baby boomers (*n* = 4189)		Earlier cohort (*n* = 2335)		Baby boomers (*n* = 7957)		Later cohort (*n* = 7720)		
	n/M	%/SD	n/M	%/SD	n/M	%/SD	n/M	%/SD	
Age at admission, years	57.0	1.8	59.4	2.1	43.7	2.0	41.4	2.0
*Sex*								
Men	2924	69.8	1542	66.0	5620	70.6	5703	73.9
Women	1265	30.2	793	34.0	2337	29.4	2017	26.1
Stable relationship with a partner	1784	42.6	1112	47.6	3198	40.2	3205	41.5
At least middle school education*	2912	69.5	1654	70.8	4898	61.6	4367	56.6
Primary AUD	3276	78.2	1994	85.4	4925	61.9	3733	48.4
*Primary ISUD*	579	13.8	163	7.0	2517	31.6	3219	41.7
Thereof opioids	487	11.6	144	6.2	1864	23.4	2067	26.8
Thereof cannabis	59	1.4	7	0.3	285	3.6	509	6.6
Thereof cocaine	22	0.5	11	0.5	283	3.6	422	5.5
Thereof stimulants	7	0.2	1	0.0	71	0.9	205	2.7
Thereof hallucinogens	1	0.0	0	0.0	1	0.0	1	0.0
Thereof volatile solvents	0	0.0	0	0.0	2	0.0	2	0.0
Thereof other psychotropic substances	3	0.1	0	0.0	11	0.1	13	0.2
Primary sedatives/hypnotics use disorder	67	1.6	38	1.6	81	1.0	69	0.9
Primary tobacco use disorder	68	1.6	50	2.1	44	0.6	49	0.6
Primary eating disorder	2	0.1	0	0.0	9	0.1	4	0.1
Primary pathological gambling	112	2.7	33	1.4	172	2.2	359	4.7
Without primary diagnosis, but specified why	85	2.0	57	2.4	209	2.6	287	3.7
Number of CUDs	1.5	1.0	1.3	0.7	1.8	1.3	1.9	1.5
Number of contacts during treatment	9.4	12.9	9.5	12.6	10.2	15.1	10.2	15.6
Utilisation of addiction care ever before	3163	75.7	1735	74.6	5961	75.2	5603	72.9

*Note*. The primary diagnosis is the addiction‐related diagnosis an individual sought help for. *Upper secondary school certificate or higher. AUD, alcohol use disorder; CUD, comorbid substance use disorders; ISUD, illicit substance use disorder.

In subsample 2, 50.8% of the 15 677 cases were baby boomers, and 29.4% of the baby boomers were female compared with 26.1% in the later cohort. AUD was the most frequent primary diagnosis presented in 61.9% of the baby boomers and 48.4% of the later cohort. Baby boomers were diagnosed with 1.8 CUDs (*SD* = 1.3) and the later cohort had on average 1.9 diagnoses (*SD* = 1.5) (Table 1, Figure 1).

### Two‐level random intercept logistic and Poisson regression models

Compared with the earlier cohort, baby boomers showed significantly lower odds for AUD [odds ratio (OR) = 0.79, 95% confidence interval (CI) = 0.66, 0.95] and, compared with the later cohort, they showed higher odds (OR = 1.50, CI = 1.37, 1.64). In subsample 2 across cohorts, women had significantly higher odds for AUD than men (OR = 1.65, CI = 1.53, 1.78). In subsample 1, there was no significant sex effect for AUD (Table 2).

**Table 2:** Random intercept logistic regression models of predictors of AUD

	Subsample 1: Baby boomers vs. earlier cohort		Subsample 2: Baby boomers vs. later cohort	
	OR (95% CI)	*P*	OR (95% CI)	*P*
*Fixed effects estimates*				
Intercept	2.64 (2.11, 3.32)	<0.001	0.57 (0.51, 0.63)	<0.001
Cohorts				
Earlier/later cohort	1.00		1.00	
Baby boomers	0.79 (0.66, 0.95)	0.014	1.50 (1.37, 1.64)	<0.001
Sex				
Men	1.00		1.00	
Women	0.92 (0.80, 1.05)	0.223	1.65 (1.53, 1.78)	<0.001
Number of contacts during treatment	1.01 (1.00, 1.02)	0.001	0.99 (0.99, 1.00)	<0.001
School education				
Low	1.00		1.00	
At least middle	2.34 (2.06, 2.67)	<0.001	2.42 (2.27, 2.59)	<0.001
Relationship with a partner				
Not stable	1.00		1.00	
Stable	1.20 (1.05, 1.37)	0.006	0.99 (0.93, 1.06)	0.769
*Random intercept estimates*				
Estimated residual variance	0.06		0.01	
Estimated residual intraclass correlations	0.02		0.00	
Observations	6524		15 677	
BIC	6176.85		20 358.35	

Note. low = lower secondary school certificate or less; at least middle = upper secondary school certificate or higher; not stable = being single, having a temporary relationship or other forms of relationships (not stable); stable = having a stable relationship with a partner. BIC, Bayesian information criterion; CI, confidence interval; OR, odds ratio.

Compared with the earlier cohort, baby boomers were more likely to present ISUD (OR = 1.58, CI = 1.24, 2.03, see Table 3) and compared with the later cohort, baby boomers were less likely to present ISUD (OR = 0.67, CI = 0.62, 0.72). In subsample 1 (OR = 0.63, CI = 0.52, 0.76) and subsample 2 (OR = 0.58, CI = 0.53, 0.63), women had lower odds for ISUD than men.

**Table 3:** Random intercept logistic regression models of predictors of ISUD

	Subsample 1: Baby boomers vs. earlier cohort		Subsample 2: Baby boomers vs. later cohort	
	OR (95% CI)	*P*	OR (95% CI)	*P*
*Fixed effects estimates*				
Intercept	0.22 (0.17, 0.29)	<0.001	1.25 (1.16, 1.34)	<0.001
Cohorts				
Earlier/Later cohort	1.00		1.00	
Baby boomers	1.58 (1.24, 2.03)	<0.001	0.67 (0.62, 0.72)	<0.001
Sex				
Men	1.00		1.00	
Women	0.63 (0.52, 0.76)	<0.001	0.58 (0.53, 0.63)	<0.001
Number of contacts during treatment			1.02 (1.01, 1.02)	<0.001
School education				
Low	1.00		1.00	
At least middle	0.34 (0.29, 0.40)	<0.001	0.38 (0.36, 0.41)	<0.001
Relationship with a partner				
Not stable	1.00		1.00	
Stable	0.63 (0.53, 0.75)	<0.001	0.87 (0.82, 0.94)	<0.001
*Random intercept estimates*				
Estimated residual variance	0.06		0.00	
Estimated residual intraclass correlations	0.02		0.00	
Observations	6524		15 677	
BIC	4318.38		19 251.80	

*Note*. low, lower secondary school certificate or less; at least middle = upper secondary school certificate or higher; not stable = being single, having a temporary relationship or other forms of relationships (not stable); stable = having a stable relationship with a partner. BIC, Bayesian information criterion; CI, confidence interval; OR, odds ratio.

SA2 targeted at distinct illicit substances (opioids, cannabis and stimulants/cocaine as outcomes) confirmed these results (see Tables S1–S3 in the Supporting Information). There were two differences in the distinct substances analysis when compared to the overall ISUD model in subsample 1. First, for primary opioids use disorder there was a sex‐specific cohort effect: in women, baby boomers had lower odds than the earlier cohort and in men, the baby boomers had higher odds than the earlier cohort. Second, regarding primary stimulants/cocaine use disorder, the cohort effect was not significant.

Regarding the number of CUDs (Table 4) a significant effect of cohort membership was observed in both subsamples: compared with the earlier cohort, the rate of CUDs was higher in baby boomers (incidence rate ratio 1.10, CI = 1.06, 1.15) and, compared with the later cohort the rate was lower for the baby boomer cohort (incidence rate ratio 0.94, CI = 0.91, 0.96).

**Table 4:** Random intercept Poisson regression models of predictors of the number of CUDs

	Subsample 1: Baby boomers vs. earlier cohort		Subsample 2: Baby boomers vs. later cohort	
	IRR (95% CI)	*P*	IRR (95% CI)	*P*
*Fixed effects estimates*				
Intercept	1.17 (1.09, 1.25)	<0.001	1.43 (1.38, 1.49)	<0.001
Cohorts				
Earlier/Later cohort	1.00		1.00	
Baby boomers	1.10 (1.06, 1.15)	<0.001	0.94 (0.91, 0.96)	<0.001
Sex				
Men	1.00		1.00	
Women	0.97 (0.92, 1.01)	0.167	0.97 (0.95, 1.00)	0.051
Number of contacts during treatment	1.00 (1.00, 1.00)	<0.001	1.00 (1.00, 1.00)	<0.001
School education				
Low	1.00		1.00	
At least middle	0.97 (0.92, 1.01)	0.142	0.96 (0.93, 0.98)	<0.001
Relationship with a partner				
Not stable	1.00		1.00	
Stable	0.95 (0.91, 0.99)	0.019	0.95 (0.93, 0.97)	<0.001
Primary diagnosis (use disorders)				
Alcohol	1.00		1.00	
Opioids	1.87 (1.77, 1.98)	<0.001	1.86 (1.81, 1.91)	<0.001
Cannabis	1.61 (1.37, 1.91)	<0.001	1.38 (1.31, 1.45)	<0.001
Cocaine and stimulants	1.73 (1.41, 2.12)	<0.001	1.54 (1.47, 1.61)	<0.001
Other psychotropic substances	1.12 (1.00, 1.26)	0.051	1.26 (1.15, 1.38)	<0.001
Pathological gambling and eating disorders	0.41 (0.33, 0.51)	<0.001	0.36 (0.32, 0.40)	<0.001
Without, but specified why	0.08 (0.05, 0.13)	<0.001	0.13 (0.11, 0.16)	<0.001
Utilisation of addiction care ever before	1.11 (1.05, 1.16)	<0.001	1.15 (1.12, 1.19)	<0.001
*Random intercept estimates*				
Estimated residual variance	0.00		0.00	
Observations	6505		15 614	
BIC	16 048.49		45 346.40	

*Note*. low = lower secondary school certificate or less; at least middle = upper secondary school certificate or higher; not stable = being single, having a temporary relationship or other forms of relationships (not stable); stable = having a stable relationship with a partner; other psychotropic substances = includes sedatives/hypnotics, hallucinogens, tobacco, volatile solvents and other psychotropic substances; BIC = Bayesian information criterion; CI = confidence interval; IRR = incidence rate ratio.

SA1 including the whole age range instead of overlapping age groups confirmed the direction of cohort differences obtained in the main analysis (see Tables S4–S7).

## Discussion

The results indicate that baby boomers in the examined Berlin outpatient addiction care facilities differed from both, an earlier and a later cohort. They were less likely to have AUD compared with the earlier cohort and more likely compared with the later cohort. Moreover, baby boomers were more likely to have ISUD and presented a higher number of CUDs compared with the earlier cohort, but less likely compared with the later cohort. These two‐way comparisons indicate that developments set off by the baby boomers regarding the investigated outcomes continue in the later cohort.

Up to now, AUD has been the most frequent primary diagnosis in the German Addiction Care Statistical Service [13]. In line with our hypothesis, seeking help for problems with illicit substances is on the rise in more recent cohorts. These findings are consistent with previous studies in other mainly high‐income countries in relation to consumption rates and admissions to addiction care [9, 10, 11, 21]. This implies that cohort‐specific developments in substance use socialisation and addiction care utilisation might be rather general in these countries. Another explanation could be that conditions of poorer health care in the earlier cohort affected life expectancy detrimentally. Therefore, for example, heroin users did not survive long enough to be present in our earlier cohort. Based on the present evidence, outpatient addiction care will have to shift its focus and expertise on illicit substances and the specific health and life situation of this clientele.

No sex‐specific cohort effects (interaction of sex*cohort) on AUD and ISUD were found, which suggests that cohort‐specific effects are not temporally shifted in women. Here, it should be considered that a narrowing sex gap in consumption and substance use disorder prevalence does not automatically affect addiction care utilisation. Women‐specific barriers to addiction care are well known [28, 29]. For example, women are more likely to seek help in non‐addiction‐specific services, as found in a US study [30]. In the case of ISUD, the lack of a sex‐specific cohort effect might also result from the relatively small number of women with ISUD, especially in the earlier cohort (subsample with earlier cohort: baby boomers *n* = 109; earlier cohort *n* = 49). Indeed, for opioids use disorder SA1 suggests a sex‐specific cohort effect in subsample 1. Female baby boomers had lower odds for opioids use disorder than women in the earlier cohort. The opposite applied to men, which is in line with the overall ISUD model. Hence, further research in samples with a higher share of women is needed to soundly judge whether sex‐specific cohort effects remain absent or are only valid for specific substances.

In comparison with the earlier cohort, baby boomers showed a higher average number of CUDs and the later cohort presented an even higher rate. This confirms the assumption of later cohorts showing a combination of multiple substance use disorders more often than earlier ones. Individuals with at least two substance‐related problems are characterised by higher overall burden [15, 16, 18] and elevated risks associated with the synergetic effects of polydrug use [15]. This is even more important as the baby boomers get older and prescription drug intake may increase, even more in case of multimorbidity [31]. To handle this issue effectively, outpatient addiction care staff need to build up expertise in case management, communication with primary care physicians and risk minimisation [16, 32, 33].

The presented results should be interpreted against some study‐related caveats. First, generalising the results to Germany as a whole is a sensitive issue. Some differences have been reported between individuals presenting with substance use disorder in outpatient addiction care in Berlin and elsewhere in Germany [34]. For instance, problems with illicit substances as well as multiple substance‐related disorders have been found to be more prevalent in Berlin than in Bavaria, a more rural region [34, 35]. Despite these regional differences, overall, similar cohort‐specific substance use socialisation processes and cohort differences are expected in rural and other urban areas. However, regarding the particular situation of Berlin during the cold war, replication studies in other German regions are needed. Second, there is evidence of an underreporting of co‐occurring mental disorders in the documentation of outpatient addiction care services resulting from structural conditions, such as staff not being sufficiently trained in diagnosing co‐occurring disorders [36]. This may also affect the reporting of comorbid substance use disorders. Based on the assumption that this underreporting occurs at random and is hence not systematically related to cohort and age, the cohort comparisons may be considered valid, even though the true prevalence level of CUDs is assumed to be higher. Third, unknown differences in the assessment of substance‐related diagnoses (e.g. unstructured vs. standardised clinical interview) may affect the reliability of the data. As the distribution of the diagnoses is similar in distinct years, this issue is compensated within the sample. Moreover, other outpatient facilities offering, for instance, predominantly psychotherapy should be analysed in future studies.

Despite these drawbacks, the study design offers some important advantages. With its large sample size, the study represents a nearly complete survey of individuals seeking help for addiction‐related problems in the Berlin outpatient addiction care system. This comprehensiveness combined with virtually free and unrestricted access to health care in Germany minimises the potential of selection and utilisation bias of point estimates. Second, using complex multilevel modelling and comparing the cohorts in overlapping age groups, it was possible to estimate crude cohort effects that are not masking time changes or age effects. When we analysed the cohorts across all age ranges at hand (SA1) instead of analysing two cohorts in overlapping age groups, cohort differences pointed in the same direction. This suggests that our findings are robust in a wider age range and that the observed differences are not restricted to single age groups. Finally, when analysing illicit substances opposite effects regarding distinct illicit substances might overlie each other. To investigate whether there was a consistent effect in the pooled sample of illicit substances, SA2 disentangled this pooled group into its most frequent underlying single substances (opioids, cannabis, stimulants/cocaine). These analyses unveiled similar patterns as the pooled analyses, suggesting that the observed trends are generalisable to illicit substances as a whole.

## Conclusion

Baby boomers were more likely to seek help because of ISUD and presented a higher number of CUDs than the earlier cohort. The later cohort showed ISUD and CUDs even more often than the baby boomers. This clearly suggests a continuing trend that is anticipated to proceed into later cohorts. In consequence, outpatient addiction care firstly needs to adapt to the gradually changing substance‐use characteristics of its clientele including multiple substance use disorders. Second, with the increased life expectancy and high substance use of the comparatively large cohort of baby boomers, outpatient addiction care needs to prepare for a growing number of older clients who had been underrepresented in addiction care so far. Evidence‐based treatment offers for this group are still scarce [37, 38] and should be expanded. The growing presence of an older clientele also implies putting more emphasis on harm reduction, tertiary prevention and well‐being [32, 33] and including geriatric approaches with patient‐centred aims such as maintaining independence or symptom management [39].

## Conflicts of Interest

SS, BB‐M, LS, N‐NS and MW declare that they have no conflict of interests. DP and LK declare having received a grant from Lundbeck GmbH for a research project on alcohol epidemiology not related to this study.

## Supporting information

**Table S1.** Random intercept logistic regression models of predictors of opioids use disorder (with interaction between sex and cohort in subsample 1 and without interaction in subsample 2).
**Table S2**. Random intercept logistic regression models of predictors of cannabis use disorder.
**Table S3**. Random intercept logistic regression models of predictors of stimulants and cocaine use disorder.
**Table S4**. Cohort characteristics in whole‐age‐range sample.
**Table S5**. Random intercept logistic regression model of predictors of AUD with interaction between sex and cohort in whole‐age‐range sample.
**Table S6**. Random intercept logistic regression model of predictors of ISUD in whole‐age‐range sample.
**Table S7**. Random intercept Poisson regression model of predictors of the number of CUDs in whole‐age‐range sample.
