# Supplementary material for: Substance use disorder and the baby boom generation: Does Berlin outpatient addiction care face a sustained change?
Source: Drug Alcohol Rev. 2021 Jan 28;40(6):979–88. doi: 10.1111/dar.13245 (PMC8451895; doi:10.1111/dar.13245)
Supplement: Supplementary file 1 — Table S1. Random intercept logistic regression models of predictors of opioids use disorder (with interaction between sex and cohort in subsample 1 and without interaction in subsample 2). Table S2. Random intercept logistic regression models of predictors of cannabis use disorder. Table S3. Random intercept logistic regression models of predictors of stimulants and cocaine use disorder. Table S4. Cohort characteristics in whole‐age‐range sample. Table S5. Random intercept logistic regression model of predictors of AUD with interaction between sex and cohort in whole‐age‐range sample. Table S6. Random intercept logistic regression model of predictors of ISUD in whole‐age‐range sample. Table S7. Random intercept Poisson regression model of predictors of the number of CUDs in whole‐age‐range sample. [file DAR-40-979-s001.docx]

**Supplemental Tables**

Table S1. Random intercept logistic regression models of predictors of opioids use disorder (with interaction between sex and cohort in subsample 1 and without interaction in subsample 2).

|  | Subsample 1: Baby boomers vs. earlier cohort | |  | Subsample 2: Baby boomers vs. later cohort | |
| --- | --- | --- | --- | --- | --- |
|  | *OR* (95% *CI*) | *P* |  | *OR* (95% *CI*) | *P* |
| **Fixed effects estimates** |  |  |  |  |  |
| Intercept | 0.17 (0.13, 0.22) | <0.001 |  | 0.62 (0.57, 0.69) | <0.001 |
| *Interactions sex * cohort* |  |  |  |  |  |
| Women * earlier cohort | 1.00 |  |  |  |  |
| Women * baby boomers | 0.58 (0.37, 0.89) | 0.013 |  |  |  |
| *Cohorts* |  |  |  |  |  |
| Earlier/Later cohort | 1.00 |  |  | 1.00 |  |
| Baby boomers | 1.91 (1.42, 2.58) | <0.001 |  | 0.83 (0.75, 0.91) | <0.001 |
| *Sex* |  |  |  |  |  |
| Men | 1.00 |  |  | 1.00 |  |
| Women | 1.00 (0.70, 1.44) | 0.984 |  | 0.75 (0.68, 0.82) | <0.001 |
| Number of contacts during treatment |  |  |  | 1.02 (1.02, 1.02) | <0.001 |
| *School education* |  |  |  |  |  |
| Low | 1.00 |  |  | 1.00 |  |
| At least middle | 0.32 (0.27, 0.38) | <0.001 |  | 0.34 (0.31, 0.36) | <0.001 |
| *Relationship with a partner* |  |  |  |  |  |
| Not stable | 1.00 |  |  | 1.00 |  |
| Stable | 0.59 (0.49, 0.71) | <0.001 |  | 0.81 (0.75, 0.87) | <0.001 |
| **Random intercept estimates** |  |  |  |  |  |
| Estimated residual variance | 0.03 |  |  | 0.01 |  |
| Estimated residual intraclass correlations | 0.01 |  |  | 0.00 |  |
| Observations | 6524 |  |  | 15,677 |  |
| BIC | 3886.00 |  |  | 16,509.96 |  |

*Note*. low = lower secondary school certificate or less; at least middle = upper secondary school certificate or higher; not stable = being single, having a temporary relationship or other forms of relationships (not stable); stable = having a firm relationship with a partner. BIC = Bayesian information criterion*;* CI = confidence interval; OR = odds ratio.

Table S2. Random intercept logistic regression models of predictors of cannabis use disorder.

|  | Subsample 1: Baby boomers vs. earlier cohort | |  | Subsample 2: Baby boomers vs. later cohort | |
| --- | --- | --- | --- | --- | --- |
|  | *OR* (95% *CI*) | *P* |  | *OR* (95% *CI*) | *P* |
| **Fixed effects estimates** |  |  |  |  |  |
| Intercept | 0.00 (0.00, 0.01) | <0.001 |  | 0.09 (0.08, 0.10) | <0.001 |
| *Cohorts* |  |  |  |  |  |
| Earlier/Later cohort | 1.00 |  |  | 1.00 |  |
| Baby boomer | 4.60 (2.10, 10.09) | <0.001 |  | 0.53 (0.46, 0.62) | <0.001 |
| *Sex* |  |  |  |  |  |
| Men |  |  |  | 1.00 |  |
| Women |  |  |  | 0.65 (0.55, 0.78) | <0.001 |
| Number of contacts during treatment |  |  |  | 0.99 (0.99, 1.00) | 0.023 |
| *Relationship with a partner* |  |  |  |  |  |
| Not stable | 1.00 |  |  | 1.00 |  |
| Stable | 0.49 (0.28, 0.85) | 0.010 |  | 0.83 (0.71, 0.96) | 0.013 |
| **Random intercept estimates** |  |  |  |  |  |
| Estimated residual variance | 0.00 |  |  | 0.00 |  |
| Estimated residual intraclass correlations | 0.00 |  |  | 0.00 |  |
| Observations | 6524 |  |  | 15,677 |  |
| BIC | 734.63 |  |  | 6,219.06 |  |

*Note*. low = lower secondary school certificate or less; at least middle = upper secondary school certificate or higher; not stable = being single, having a temporary relationship or other forms of relationships (not stable); stable = having a firm relationship with a partner; BIC = Bayesian information criterion*;* CI = confidence interval; OR = odds ratio.

Table S3. Random intercept logistic regression models of predictors of stimulants and cocaine use disorder.

|  | Subsample 1: Baby boomers vs. earlier cohort | |  | Subsample 2: Baby boomers vs. later cohort | |
| --- | --- | --- | --- | --- | --- |
|  | *OR* (95% *CI*) | *P* |  | *OR* (95% *CI*) | *P* |
| **Fixed effects estimates** |  |  |  |  |  |
| Intercept | 0.01 (0.00, 0.02) | <0.001 |  | 0.10 (0.08, 0.12) | <0.001 |
| *Cohorts* |  |  |  |  |  |
| Earlier/Later cohort | 1.00 |  |  | 1.00 |  |
| Baby boomers | 1.27 (0.60, 2.73) | 0.532 |  | 0.63 (0.52, 0.77) | <0.001 |
| *Sex* |  |  |  |  |  |
| Men | 1.00 |  |  | 1.00 |  |
| Women | 0.18 (0.06, 0.60) | 0.005 |  | 0.37 (0.31, 0.45) | <0.001 |
| *School education* |  |  |  |  |  |
| Low | 1.00 |  |  | 1.00 |  |
| At least middle | 0.47 (0.25, 0.89) | 0.019 |  | 0.77 (0.67, 0.87) | <0.001 |
| *Relationship with a partner* |  |  |  |  |  |
| Not stable | 1.00 |  |  | 1.00 |  |
| Stable | 2.25 (1.18, 4.29) | 0.013 |  | 1.33 (1.16, 1.51) | <0.001 |
| **Random intercept estimates** |  |  |  |  |  |
| Estimated residual variance | 0.04 |  |  | 0.02 |  |
| Estimated residual intraclass correlations | 0.01 |  |  | 0.01 |  |
| Observations | 6524 |  |  | 15,677 |  |
| BIC | 525.33 |  |  | 7138.79 |  |

*Note*. low = lower secondary school certificate or less; at least middle = upper secondary school certificate or higher; not stable = being single, having a temporary relationship or other forms of relationships (not stable); stable = having a stable relationship with a partner; BIC = Bayesian information criterion*;* CI = confidence interval; OR = odds ratio.

**Sensitivity analyses comparing baby boomers with the earlier and later cohort in all available age groups (whole-age-range sample)**

Table S4. Cohort characteristics in whole-age-range sample.

| Characteristics | Baby boomers (*n* = 25,835) | |  | Earlier cohort (*n* = 4667) | |  | Later cohort (*n* = 33,111) | |  |
| --- | --- | --- | --- | --- | --- | --- | --- | --- | --- |
|  | *n/M* | *%/SD* |  | *n/M* | *%/SD* |  | *n/M* | *%/SD* |  |
| Age at admission, years | 49.3 | 4.9 |  | 63.1 | 4.7 |  | 34.3 | 5.2 |  |
| *Sex* |  |  |  |  |  |  |  |  |  |
| Men | 18,012 | 69.7 |  | 2888 | 61.9 |  | 24,889 | 75.2 |  |
| Women | 7823 | 30.3 |  | 1779 | 38.1 |  | 8222 | 24.8 |  |
| Stable relationship with a partner | 10,544 | 40.8 |  | 2272 | 48.7 |  | 13,414 | 40.5 |  |
| At least middle school education | 17,007 | 65.8 |  | 3236 | 69.3 |  | 16,882 | 51.0 |  |
| Primary AUD | 17,930 | 69.4 |  | 3952 | 84.7 |  | 12,074 | 36.5 |  |
| *Primary ISUD* | 6005 | 23.2 |  | 253 | 5.4 |  | 18,066 | 54.6 |  |
| Thereof opioids | 4672 | 18.1 |  | 216 | 4.6 |  | 9460 | 28.6 |  |
| Thereof cannabis | 644 | 2.5 |  | 16 | 0.3 |  | 4845 | 14.6 |  |
| Thereof cocaine | 501 | 1.9 |  | 17 | 0.4 |  | 2311 | 7.0 |  |
| Thereof stimulants | 162 | 0.6 |  | 3 | 0.1 |  | 1357 | 4.1 |  |
| Thereof hallucinogens | 3 | 0.0 |  | 0 | 0.0 |  | 12 | 0.0 |  |
| Thereof volatile solvents | 2 | 0.0 |  | 0 | 0.0 |  | 17 | 0.1 |  |
| Thereof other psychotropic substances | 21 | 0.1 |  | 1 | 0.0 |  | 64 | 0.2 |  |
| Primary sedatives/hypnotics use disorder | 302 | 1.2 |  | 118 | 2.5 |  | 246 | 0.7 |  |
| Primary tobacco use disorder | 217 | 0.8 |  | 122 | 2.6 |  | 134 | 0.4 |  |
| Primary eating disorder | 21 | 0.1 |  | 1 | 0.0 |  | 15 | 0.1 |  |
| Primary pathological gambling | 676 | 2.6 |  | 118 | 2.5 |  | 1256 | 3.8 |  |
| Without primary diagnosis, but specified why | 684 | 2.7 |  | 103 | 2.2 |  | 1320 | 4.0 |  |
| Number of CUDs | 1.6 | 1.2 |  | 1.2 | 0.6 |  | 2.1 | 1.6 |  |
| Number of contacts during treatment | 9.7 | 14.1 |  | 9.5 | 12.4 |  | 9.8 | 14.8 |  |
| Utilisation of addiction care ever before | 19,535 | 75.9 |  | 3407 | 73.3 |  | 22,253 | 67.5 |  |

*Note*. At least middle school education = upper secondary school certificate or higher; The primary diagnosis is the addiction-related diagnosis an individual sought help for. AUD = alcohol use disorder; CUD = comorbid substance use disorders; ISUD = illicit substance use disorder.

Table S5. Random intercept logistic regression model of predictors of AUD with interaction between sex and cohort in whole-age-range sample.

|  | *OR* (95% *CI*) | *P* |
| --- | --- | --- |
| **Fixed effects estimates** |  |  |
| Intercept | 0.66 (0.60, 0.72) | <0.001 |
| *Interactions sex * cohort* |  |  |
| Women * baby boomers | 1.00 |  |
| Women * earlier cohort | 0.57 (0.48, 0.68) | <0.001 |
| Women * later cohort | 1.16 (1.07, 1.26) | <0.001 |
| *Cohorts* |  |  |
| Baby boomers | 1.00 |  |
| Earlier cohort | 1.80 (1.57, 2.07) | <0.001 |
| Later cohort | 0.41 (0.39, 0.43) | <0.001 |
| *Sex* |  |  |
| Men | 1.00 |  |
| Women | 1.30 (1.22, 1.38) | <0.001 |
| *Age at admission, years* |  |  |
| Up to 35 | 1.00 |  |
| 36-55 | 1.89 (1.80, 1.98) | <0.001 |
| 56+ | 3.27 (2.95, 3.63) | <0.001 |
| Number of contacts during treatment | 1.00 (0.99, 1.00) | <0.001 |
| *School education* |  |  |
| Low | 1.00 |  |
| At least middle | 2.16 (2.09, 2.24) | <0.001 |
| *Relationship with a partner* |  |  |
| Not stable | 1.00 |  |
| Stable | 1.04 (1.00, 1.08) | 0.035 |
| **Random intercept estimates** |  |  |
| Estimated residual variance | 0.01 |  |
| Estimated residual intraclass correlations | 0.00 |  |
| Observations | 63,613 |  |
| BIC | 75,987.24 |  |

*Note*. low = lower secondary school certificate or less; at least middle = upper secondary school certificate or higher; not stable = being single, having a temporary relationship or other forms of relationships (not stable); stable = having a stable relationship with a partner, BIC = Bayesian information criterion*;* CI = confidence interval; OR = odds ratio.

Table S6. Random intercept logistic regression model of predictors of ISUD in whole-age-range sample.

|  | *OR* (95% *CI*) | *P* |
| --- | --- | --- |
| **Fixed effects estimates** |  |  |
| Intercept | 1.00 (0.93, 1.08) | 0.947 |
| *Cohorts* |  |  |
| Baby boomers | 1.00 |  |
| Earlier cohort | 0.35 (0.30, 0.42) | <0.001 |
| Later cohort | 2.46 (2.35, 2.58) | <0.001 |
| *Sex* |  |  |
| Men | 1.00 |  |
| Women | 0.69 (0.66, 0.72) | <0.001 |
| *Age at admission, years* |  |  |
| Up to 35 | 1.00 |  |
| 36-55 | 0.56 (0.53, 0.58) | <0.001 |
| 56+ | 0.28 (0.25, 0.32) | <0.001 |
| Number of contacts during treatment | 1.01 (1.01, 1.01) | <0.001 |
| *School education* |  |  |
| Low | 1.00 |  |
| At least middle | 0.43 (0.41, 0.44) | <0.001 |
| *Relationship with a partner* |  |  |
| Not stable | 1.00 |  |
| Stable | 0.85 (0.82, 0.88) | <0.001 |
| **Random intercept estimates** |  |  |
| Estimated residual variance | 0.00 |  |
| Estimated residual intraclass correlations | 0.00 |  |
| Observations | 63,613 |  |
| BIC | 71,423.83 |  |

*Note*. low = lower secondary school certificate or less; at least middle = upper secondary school certificate or higher; not stable = being single, having a temporary relationship or other forms of relationships (not stable); stable = having a stable relationship with a partner; BIC = Bayesian information criterion*;* CI = confidence interval; OR = odds ratio.

Table S7. Random intercept Poisson regression model of predictors of the number of CUDs in whole-age-range sample.

|  | *IRR* (95% *CI*) | *P* |
| --- | --- | --- |
| **Fixed effects estimates** |  |  |
| Intercept | 1.42 (1.38, 1.46) | <0.001 |
| *Cohorts* |  |  |
| Baby boomers | 1.00 |  |
| Earlier cohort | 0.91 (0.88, 0.95) | <0.001 |
| Later cohort | 1.08 (1.06, 1.10) | <0.001 |
| *Sex* |  |  |
| Men | 1.00 |  |
| Women | 0.96 (0.95, 0.97) | <0.001 |
| *Age at admission, years* |  |  |
| Up to 35 | 1.00 |  |
| 36-55 | 0.95 (0.93, 0.96) | <0.001 |
| 56+ | 0.87 (0.84, 0.90) | <0.001 |
| Number of contacts during treatment | 1.00 (1.00, 1.00) | <0.001 |
| *School education* |  |  |
| Low | 1.00 |  |
| At least middle | 0.96 (0.94, 0.97) | <0.001 |
| *Relationship with a partner* |  |  |
| Not stable | 1.00 |  |
| Stable | 0.95 (0.93, 0.96) | <0.001 |
| *Primary diagnosis (use disorders)* |  |  |
| Alcohol | 1.00 |  |
| Opioids | 1.78 (1.75, 1.81) | <0.001 |
| Cannabis | 1.29 (1.27, 1.32) | <0.001 |
| Cocaine and stimulants | 1.53 (1.50, 1.56) | <0.001 |
| Other psychotropic substances | 1.22 (1.17, 1.27) | <0.001 |
| Pathological gambling and eating disorders | 0.33 (0.31, 0.35) | <0.001 |
| Without, but specified why | 0.14 (0.13, 0.15) | <0.001 |
| Utilisation of addiction care ever before | 1.16 (1.14, 1.18) | <0.001 |
| **Random intercept estimates** |  |  |
| Estimated residual variance | 1,028,666 |  |
| Observations | 63,381 |  |
| BIC | 183,627.2 |  |

*Note*. low = lower secondary school certificate or less; at least middle = upper secondary school certificate or higher; not stable = being single, having a temporary relationship or other forms of relationships (not stable); stable = having a stable relationship with a partner; other psychotropic substances = includes sedatives/hypnotics, hallucinogens, tobacco, volatile solvents and other psychotropic substances. BIC = Bayesian information criterion*;* CI = confidence interval; IRR = incidence rate ratio.
